# Usefulness of lung ultrasound for selecting asymptomatic older patients with COVID 19 pneumonia

**DOI:** 10.1038/s41598-021-02275-2

**Published:** 2021-11-24

**Authors:** Chukwuma Okoye, Valeria Calsolaro, Alessandra Fabbri, Riccardo Franchi, Rachele Antognoli, Ludovica Zisca, Camilla Bianchi, Alessia Maria Calabrese, Sara Rogani, Fabio Monzani

**Affiliations:** 1 Geriatrics Unit, Department of Clinical & Experimental Medicine, University Hospital of Pisa, Via Paradisa, 2, 56124 Pisa, Italy; 2 Low Care Unit, Presidio Sanitario “Anna Torrigiani”, Firenze, Italy

**Keywords:** Diseases, Health care, Signs and symptoms

## Abstract

Clinical and prognostic differences between symptomatic and asymptomatic older patients with COVID-19 are of great interest since frail patients often show atypical presentation of illness. Lung Ultrasound (LUS) has been proven to be a reliable tool for detecting early-phase COVID-19 pneumonic alterations. The current prospective bicentric study aimed to compare LUS score and 3-month overall mortality between asymptomatic and symptomatic older patients with COVID-19, according to frailty status. Patients were stratified according to LUS score tertiles and Clinical Frailty Scale categories. Survival rate was assessed by telephone interviews 3 months after discharge. 64 symptomatic (24 women, aged 80.0 ± 10.8 years) and 46 asymptomatic (31 women, aged 84.3 ± 8.8 years) were consecutively enrolled. LUS score resulted an independent predictor of 3-month mortality [OR 2.27 (CI95% 1.09–4.8), p = 0.03], and the highest mortality rate was observed in symptomatic and asymptomatic pre-frail and frail patients (70.6% and 66.7%, respectively) with greater LUS abnormalities (3rd tertile). In conclusion, LUS identified an acute interstitial lung involvement in most of the older asymptomatic patients. Mortality rate progressively increased according to clinical frailty and LUS score degree, resulting a reliable prognostic tool in both symptomatic and asymptomatic patients.

## Introduction

Severe Acute Respiratory Syndrome caused by Coronavirus, etiological agent of COVID-19 associated pneumonia, has been identified all over the world since, in December 2019, SARS-CoV-2 has been isolated in China for the first time^1^. The clinical spectrum of SARS-CoV-2 ranges from asymptomatic infection to a multiorgan inflammatory disease that can degenerate in ARDS (acute respiratory distress syndrome). A major challenge to containing the spread of SARS-CoV-2 is that asymptomatic and pre-symptomatic individuals are infectious, and up to 50% of cases may be attributable to transmission from asymptomatic or pre-symptomatic people^2^. To date, few studies have sought at comparing clinical characteristics and pulmonary findings between asymptomatic and symptomatic patients^3,4^; a recent meta-analysis proved that more than half of the individuals with SARS-CoV-2 infection without any symptoms presented with CT abnormalities; however, the mean age was low (31.0 ± 23.8 years), and few cases of asymptomatic older patients were described^5^. In particular, clinical status of older patients with asymptomatic COVID-19 is of great interest, since frail patients often show non-typical COVID-19 clinical presentation; in a retrospective study by Bianchetti et al.^6^ regarding older patients with dementia, classical COVID-19 symptoms as dyspnoea or fever were recorded at ED admission in less than one half inpatients. Noteworthy, frail patients are the most at risk for poor outcome, facing a three times higher risk of in-hospital mortality compared to non-frail counterparts^7^. The Office for National Statistics (ONS) in England and Wales reported a 79% increase in total deaths in care homes from 2 March to 12 June compared to 2015–2019, higher in nursing homes than residential homes^8^. Moreover, a recent study by Rutten et al.^9^ reported that, despite symptomatology overlapped between nursing-home residents with and without COVID-19, those with COVID-19 were 3 times more likely to die within 30 days. For these reasons, early detection of the severity of pulmonary involvement is crucial in older patients with COVID-19 diagnosis, regardless their clinical presentation. The American College of Radiology recommends against the use of CT as a screening or initial imaging study to diagnose Covid-19^10^; whereas lung ultrasonography has been proven to represent a valid alternative in the early staging of the disease^11^. LUS has proven to exhibit a good concordance with HRCT^12^ by evaluating the degree of pulmonary involvement via the amount of focal or confluent B-lines and the presence of thickened pleural line with spared areas. The aim of the current study is to evaluate lung ultrasound artefacts in older patients hospitalized for symptomatic SARS-CoV-2 pneumonia, as compared to asymptomatic subjects with positive COVID-19 nasopharyngeal swab (NS). Secondary endpoint is to assess the possible relationship between LUS score and short-term mortality rate in symptomatic and asymptomatic patients.

## Methods

We enrolled all the patients older than 65 years consecutively admitted to the Geriatrics Unit of our tertiary care hospital from April 10th to December 12th 2020, with severe SARS-CoV-2 pneumonia, as defined by NIH criteria^13^. Thus, all the patients had SpO2 below 94% on room air at sea level, respiratory rate above 30 breaths/min, PaO_2_/FiO_2_ratio (i.e., partial pressure arterial oxygen/fraction of inspired oxygen ratio) below 300 mmHg, or over 50% lung infiltrates. Exclusion criteria were: (i) critical illness requiring invasive ventilation at admission, (ii) presence of concomitant pulmonary oedema at admission, (iii) pre-existing interstitial diseases. All the symptomatic patients underwent HRCT immediately before ward admission, as part of the Emergency Department (ED) evaluation route of SARS-CoV-2 pneumonia. Bedside LUS was independently performed by two skilled clinicians (C.O., D.G.) at admission (within 12 h), blinded at HRCT results.

Furthermore, we enrolled asymptomatic, older patients admitted to a COVID-19 low care unit from May 18th to December 12th, 2020. All the subjects were previously nursing home residents, temporarily transferred for observation after having two consecutive PCR nasopharyngeal swabs positive for SARS-CoV-2 infection, with a PCR cycle threshold (Ct) value ≤ 30. In this setting, all the patients met the NIH criteria for asymptomatic/pre-symptomatic SARS-CoV-2 infection^13^; hence, patients showing any respiratory symptoms, fever, or peripheral O_2_ saturation below 94% in air room during the hospital stay, were excluded from the final analysis. LUS was performed by two skilled clinicians (C.O, L.Z) within the 10th to 14th day after positive NS. At ward admission, the presence of positive history of chronic obstructive pulmonary disease, chronic heart failure, diabetes and arterial hypertension was recorded. Comprehensive geriatric assessment (CGA) was performed using the following scales: Activities of Daily Living (ADL)^14^, Instrumental ADL^15^, Short Portable Mental Status Questionnaire (SPMSQ)^16^, Cumulative Illness Rating Scale (CIRS)^17^. The presence of frailty was defined according to the Rockwood Clinical Frailty Scale^18^ as follows: patients with score 1–3 were categorized as “Robust”; those with score 4–5 as “Pre-Frail”, and those with score 6–9 as “Frail”.

In both the study settings, a convex and a linear covered probe, 3.5 to 7.5 MHz (Esaote Medical System), were used for chest ultrasound examination. For patients with severe mobility limitations, two operators were concomitantly involved, according to current guidelines^19^. As for the scanning scheme, in the absence of a standardized score for COVID-19 patients, we used a previously validated score^20^, namely LUS score for monitoring aeration, ranging from 0 to 3 points in each of the 16 scanned zones. Score 0: predominant A-lines or less than 3 separated B-lines. Score 1: at least 3 B-lines or coalescent B-lines occupying ≤ 50% of the screen without a clearly irregular pleural line. Score 1p: at least 3 B-lines or coalescent B-lines occupying ≤ 50% of the screen with a clearly irregular pleural line. Score 2: coalescent B-lines occupying > 50% of the screen without a clearly irregular pleural line. Score 2p: coalescent B-lines occupying > 50% of the screen with a clearly irregular pleural line. Score 3: large consolidations (at least > 1 cm). The final score is obtained by summing up the scores of each area and dividing the sum for the total of zone recorded (Supplemental Fig. S2). All the patients (symptomatic and asymptomatic) were categorized according to LUS score tertiles as follows: T1 (Tertile 1) = LUS score ≤ 0.5, T2 (Tertile 2) = LUS score 0.6–1.4; T3 (Tertile 3) = LUS Score ≥ 1.5. Sensitivity analysis comparing ultrasonographic findings using HRCT as gold standard was performed to confirm LUS accuracy in symptomatic patients.

Each patient of the two cohorts gave written informed consent to participate in the study; in case of patient’s inability, the legally authorized delegate provided informed consent. The study protocol complied with the Declaration of Helsinki and was approved by the local Ethic Committee (Comitato Etico Regionale per la Sperimentazione Clinica della Regione Toscana, Area Nord Ovest (CEAVNO) no protocol: CEAVNO-2020-17241). Three-month mortality rate of both symptomatic and asymptomatic patients was assessed by telephone interview.

### Statistical analysis

Statistical analysis was performed by using SPSS 27.0 statistical software package (SPSS Inc., Chicago, IL) and GraphPad Prism 9. Continuous variables were expressed as mean ± standard deviation or, for non-normally skewed distributions, as median an interquartile (IQR), and categorical data were expressed as percentage. Mann–Whitney U-test and χ^2^ exact tests were used for multiple comparisons. LUS score tertiles were considered as 3-ordinal-variables, and a Cochrane-Armitage test was used for multiple comparison for the mortality outcome, utilizing a *p* for trend. LUS diagnostic performance was assessed by calculating specificity, sensitivity, negative predictive value (NPV) and positive predictive value (PPV), using HRCT as gold standard. Proportion of agreement (P_0_) was utilized to represent the concordance between HRCT and LUS. In order to evaluate the association between LUS score and 3-months all-cause mortality, a logistic univariate regression analysis was carried out, using LUS score as continuous numerical dependent variable. Multivariate logistic regression analysis was also calculated after adjusting for age, sex, history of heart failure (HF), Chronic obstructive pulmonary disease (COPD) and obesity. Statistical significance was considered for *p* < 0.05.

### Ethical approval and consent to participate

Each patient gave written informed consent to participate in the study; in case of patient’s inability, the legally authorized delegate provided informed consent. The study protocol complied with the Declaration of Helsinki and was approved by the local ethics committee: Comitato Etico Regionale per la Sperimentazione Clinica della Regione Toscana, Area Nord Ovest (no protocol: CEAVNO-2020-17241).

## Results

**Figure 1:**
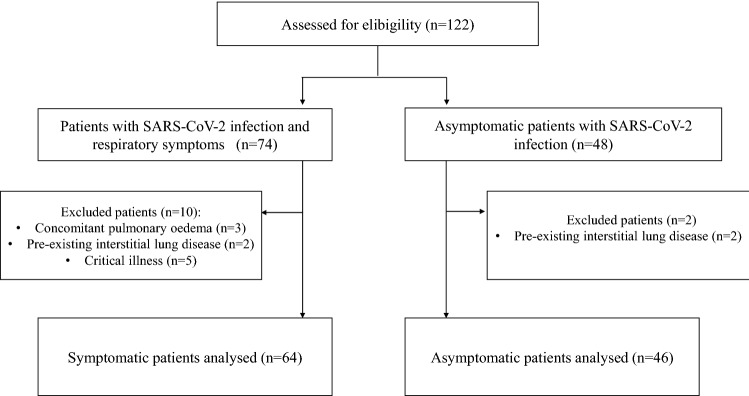
Flow chart of the observational cohort study.

A total of 122 older patients with SARS-CoV-2 infection were initially enrolled; 12 of them were subsequently excluded as described in Fig. 1. Thus, 110 patients (mean age 82.3 ± 10.1 years) were finally included into the study: 64 (59.2%) with symptomatic SARS-CoV-2 pneumonia from the acute Geriatrics ward setting and 46 (41.8%) asymptomatic subjects from the COVID-19 low-care setting. Baseline characteristics of the study population are detailed in Table 1.

**Table 1:** Clinical characteristics of symptomatic and asymptomatic older patients with SARS-CoV-2 infection.

	Whole cohort N = 110	Symptomatic N = 64	Asymptomatic N = 46	*p*
Gender (F%)	55 (50.0)	24 (37.5)	31 (67.4)	** < 0.01**
Age (years)	82.3 ± 10.2	80.8 ± 10.8	84.3 ± 8.8	0.04
ADL (median, IQR)	4 (0–6)	6 (2–6)	2 (0–6)	** < 0.01**
SPMSQ (median, IQR)	2 (0–8)	1 (0–5)	4 (0–10)	0.06
CIRS-c (mean ± SD)	1.80 ± 1.5	2.4 ± 1.4	1.4 ± 0.9	** < 0.001**
Clinical frailty scale				
*Frail (%)*	43 (39.1)	22 (34.4)	21 (45.6)	** < 0.001**
*Pre-Frail (%)*	24 (21.8)	7 (10.9)	17 (37.0)	
*Robust (%)*	43 (39.1)	35 (54.7)	8 (17.4)	
Chronic respiratory diseases (%)	16 (31.4)	7 (26.9)	9 (36)	0.48
Chronic heart failure (%)	19 (37.2)	10 (40)	9 (34.6)	0.06
Arterial hypertension (%)	32 (62.5)	18 (69.2)	14 (56)	0.18
Diabetes mellitus (%)	10 (19.7)	7 (28)	3 (11.5)	0.17
Obesity (%)	10 (19.7)	6 (24)	4 (15.3)	0.39
WBC/mm^3^ (median, IQR)	7043 (4000)	6710 (3810)	5180 (4170)	0.655
Lymphocyte count/mm^3^ (median, IQR)	1390 (780)	995 (692)	1610 (2375)	0.101
Hs-CRP mg/dl (median, IQR)	6.44(8.20)	6.09 (10.3)	2.10 (3.70)	** < 0.001**

Data are expressed as mean ± standard deviation, median (interquartile range) and number (%) as appropriate.

*ADL* activities of daily living, *CIRS-C* cumulative illness rating scale-comorbidity, *SPMSQ* short portable mental status questionnaire, *WBC* white blood cells, *Hs-CRP* high-sensitivity C-reactive protein.

Significant *p* values are marked in bold.

**Figure 2:**
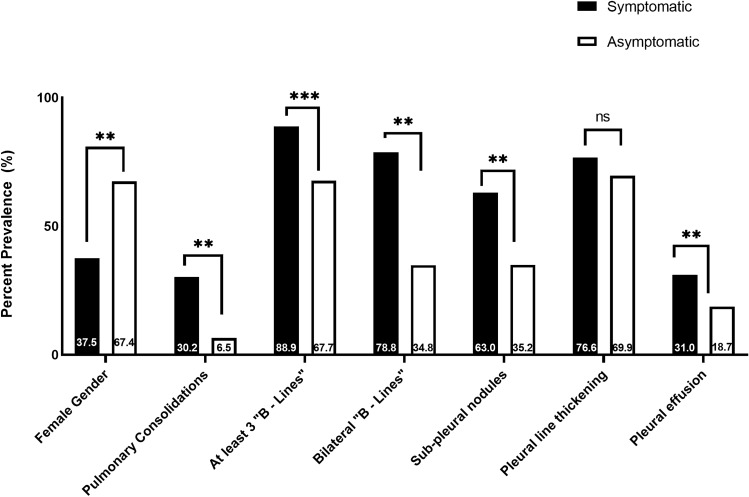
Lung ultrasound features of symptomatic and asymptomatic older patients with SARS-CoV2-infection. **p-value < 0.01, ***p-value < 0.001. *ns* not significant.

At LUS examination, at least three B-lines artefacts (78.3%) and irregular thickened pleural line with spared areas (73.6%) resulted the predominant pattern of abnormalities observed (Fig. 2). B-lines were commonly observed (88.9%), usually bilateral (78.8%), in symptomatic patients and, although less frequently (67.7%, p < 0.01 *vs* symptomatic), with a lower bilateral distribution (34.8%, p < 0.001 *vs* symptomatic) in asymptomatic patients. Pleural effusion was found in 20 (31.1%) symptomatic and 9 (18.7%) asymptomatic patients (p = 0.005).

**Figure 3:**
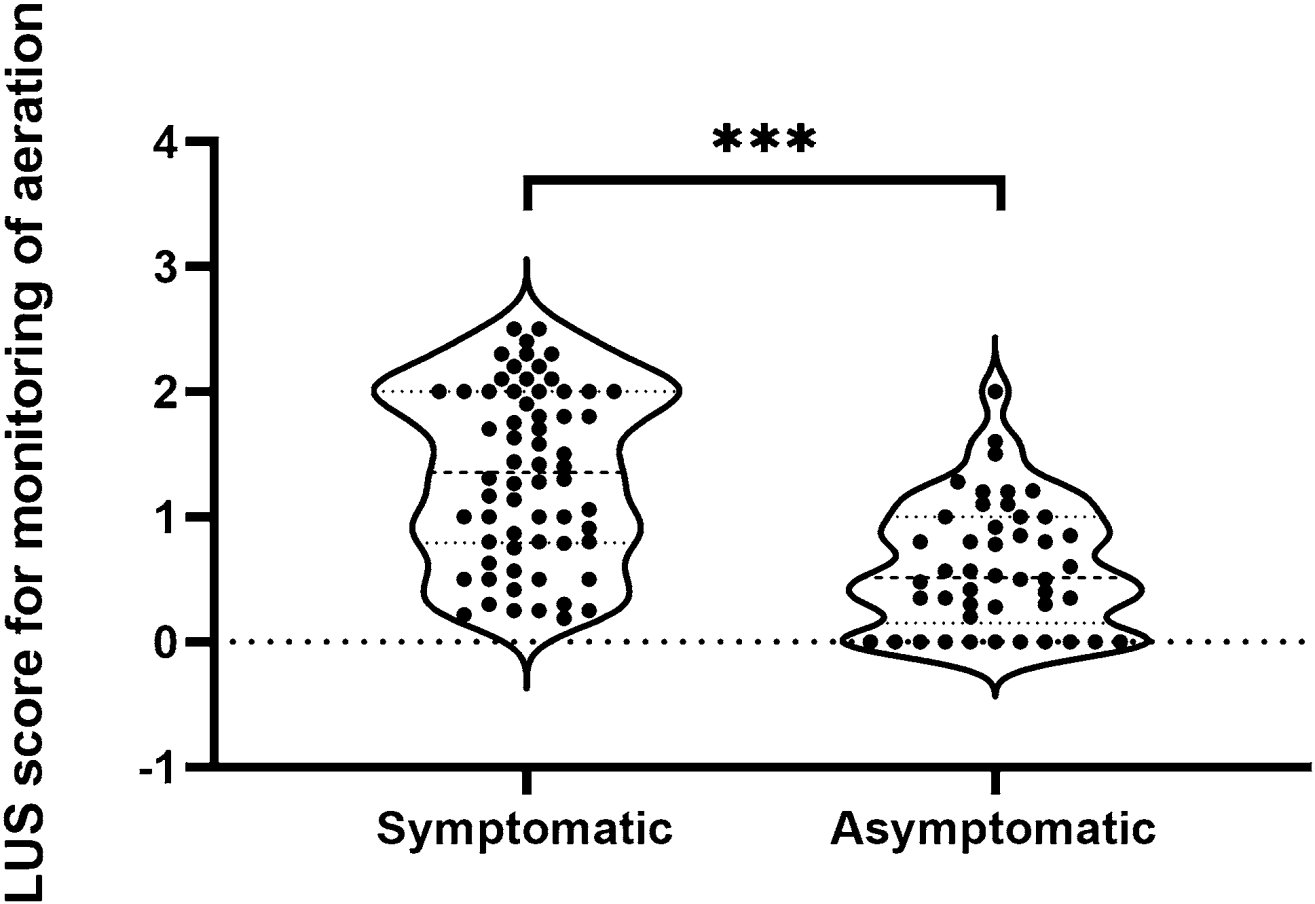
Distribution of lung ultrasound score in symptomatic and asymptomatic older patients with SARS-CoV-2 infection. ***p-value < 0.0001.

Overall, mean LUS score resulted significantly higher in symptomatic than asymptomatic patients (1.34 ± 0.69 vs 0.60 ± 0.50, p < 0.001) (Fig. 3). By sensitivity analysis, B-lines artefacts were found in 54 out of 57 (95.1%) patients with ground glass opacities at HRCT (P_o_ = 95%) with 100%sensitivity (95% CI 92.4–100%) and 40% specificity (95% CI 0.1–82.9); PPV 95.1% (95% CI 84.6–98.8) and NPV 100%], yielding a diagnostic accuracy of 95.8% (95% CI 78.9–99.9). All the cases of pulmonary consolidations and pleural effusion detected by LUS were confirmed at HRCT examination (P_o_ = 100%).

### Survival analysis

**Figure 4:**
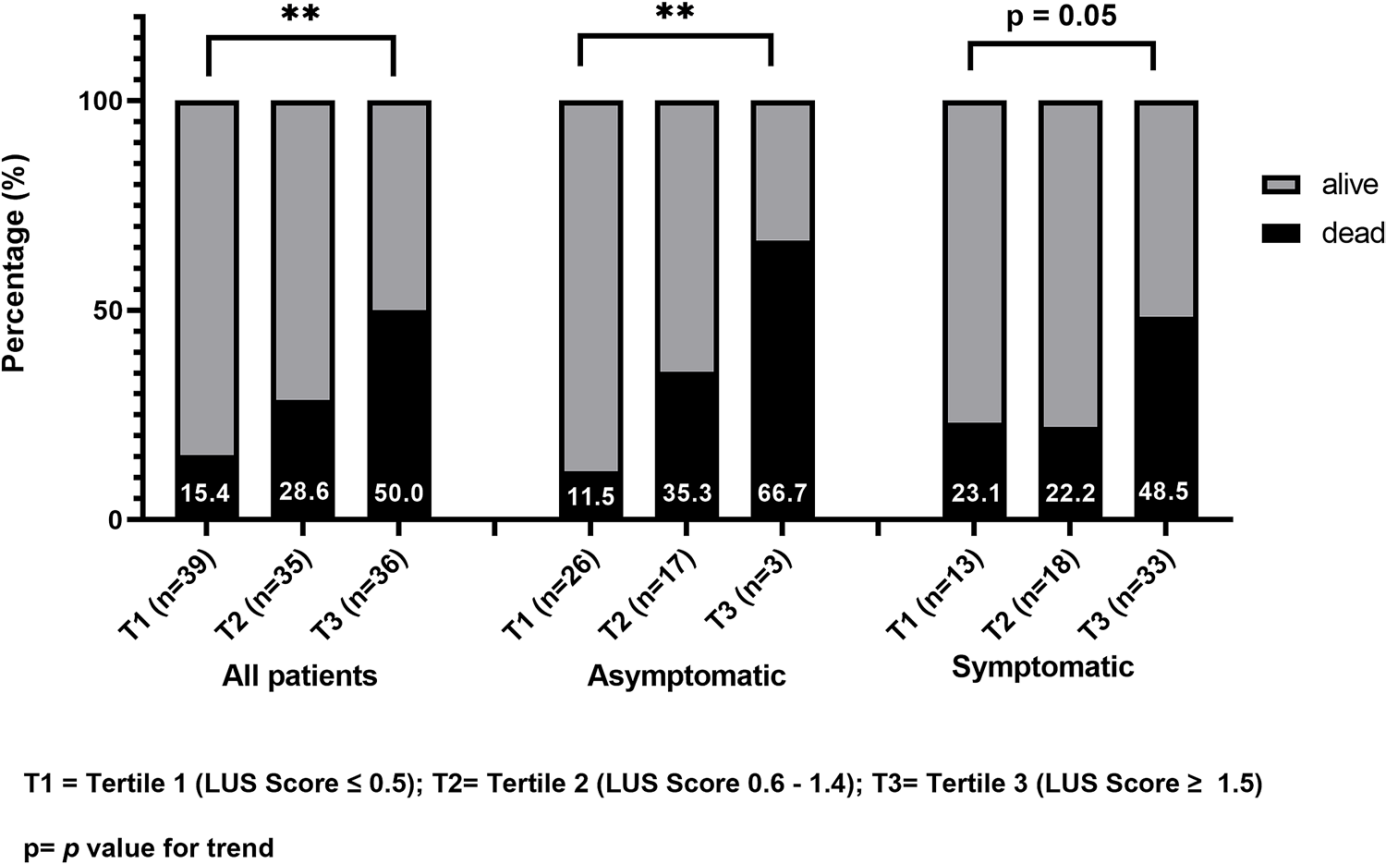
Three-months all-cause-mortality of older patients with SARS-CoV2 infection according to LUS score tertiles. **p-value < 0.01.

Nine symptomatic patients died during hospital stay; further 14 symptomatic and 11 asymptomatic patients died at 3-month follow-up. Thus, the overall mortality rate was 35.9% in symptomatic and 23.9% in asymptomatic patients (p = 0.17). Moving from the lowest to the highest LUS score tertile, a significant, progressive increase of mortality rate was observed in the whole cohort of patients (15.4%, 28.6% and 50.0%, respectively; *p* for trend = 0.001). The mortality trend remained significant among asymptomatic (11.5%, 35.3% and 66.7%, respectively; *p* for trend = 0.013) but not symptomatic patients (23.1%, 22.2% and 48.5%, respectively; *p* for trend = 0.057) (Fig. 4).

According to CFS, pre-frail and frail patients showed an increased mortality typically in the highest tertile of LUS score compared to T1 and T2, both in asymptomatic (66.7% vs 36.3% and 30%, respectively) and symptomatic patients (70.6% vs 25.0% and 28.6%), as shown in Supplemental Table S1. Furthermore, LUS score emerged as an independent predictor of 3-months all-cause mortality at multivariate analysis after adjusting for age, sex, BMI, diabetes, heart failure and COPD (unadjusted OR 2.29, CI95% 1.11–4.9, p = 0.028, adjusted OR 2.27, CI95% 1.09–4.8, p = 0.030).

## Discussion

In our study, more than two-thirds of asymptomatic older patients with SARS-CoV-2 infection showed signs of pulmonary involvement evaluated by lung ultrasound. Asymptomatic patients were more likely female, with a lower burden of comorbidity, usually showing unilateral interstitial involvement. As expected, overall mortality rate of asymptomatic patients was lower than symptomatic patients but, moving from the lowest to the highest LUS score tertile, their mortality rate showed a significant, progressive increase, typically in frail and pre-frail patients. Although older individuals suffering from COVID-19 represent the prototype of patients most at risk for adverse outcomes, to our knowledge, no previous studies have attempted to assess LUS artefacts and their relationship with a mid-term mortality risk in asymptomatic oldest patients.

It is well-established that a proportion of patients with COVID-19 without symptoms show HRCT signs of acute pneumonia; however, less is known about the prognostic impact of these findings in older frail patients, since they usually face disproportionate non-typical symptoms and an increase excess mortality after hospital discharge which is not completely understood^8,9^. In the current study we hypnotized a crucial role of asymptomatic pneumonia evaluated with LUS, in the mid-term course of the disease in this particular class of frailest individuals with SARS-CoV-2 infection.

Lung ultrasound as been proved to be an important tool for interstitial lung evaluation of COVID-19 pneumonia and in the follow-up of the disease^21^. As reported by Meroi et al. LUS is a time-saving tool compared to traditional radiology, being useful for monitoring patients’ conditions throughout their hospital stay and after discharge^22^. In COVID-19, we typically see various grades of multiple B-lines with patchy distribution. B-lines can be separated or coalescent, including pictures of sonographic ‘white lung’^23^. In agreement with Volpicelli et al.^11^, in our study at least three B lines and a fragmented pleural line were the most frequent ultrasonographic findings, both in asymptomatic and symptomatic patients. In our study, asymptomatic patients were more likely to present unilateral involvement, as first described by Shi et al.^3^, who found unilateral, multifocal ground glass opacities at Chest CT. This finding is particularly of interest in the low care hospitals and nursing homes, where an accurate risk stratification is crucial to decide which older patients need to be transferred to a hospital or could be treated in the same setting. As expected, we found a significantly higher LUS score of aeration in symptomatic patients compared to asymptomatic pairs. In other words, patients with respiratory symptoms, showed a higher number of diffuse, bilateral B-lines compared to the asymptomatic ones, thus confirming an increased interstitial pulmonary damage.

In the current study we found a high prevalence of comorbidities, especially cardiovascular and chronic respiratory diseases. Consistent with previous studies^24^, patients with severe COVID-19 manifestations showed a higher prevalence of cardiovascular disorders, compared to asymptomatic, although not reaching the statistical significance. Somewhat surprisingly, asymptomatic patients were older and frailer than those symptomatic; however, this result need to be contextualized in the clinical setting where the asymptomatic patients came from. Indeed, asymptomatic patients were residents of a nursing home temporarily transferred to a COVID-19 low care unit for clinical observation, while symptomatic patients were hospitalized in an acute care geriatrics unit. Although the prognostic role of multimorbidity and frailty is still debated^25^, as a fact, older people living in nursing homes are commonly oldest with various degree of disability, needing assistance in basic activities of daily living.

Given that older individuals may present non-specific LUS pattern, such as localized B lines or pleural thickening due to pre-existing fibrosis^26,27^, we performed a concordance analysis by estimating the correlation of LUS findings and HRCT features in the acute geriatric ward. As expected, we found a high proportion of agreement in terms of B-lines, pulmonary consolidations and pleural effusions, consistent with a recent paper by Nouvenne et al.^12^, in which the significant correlation between HRCT and LUS findings in COVID-19 was firstly reported. Notwithstanding, it is important to underline that in the current pandemic time-frame COVID-19 represents the most frequent interstitial lung disease; however, in the future, a decline of COVID-19 infection rates may reduce pre-test probability and LUS positive likelihood ratio in favor of other interstitial lung diseased with similar LUS-patterns^28^.

During the 3-month post-discharge follow-up, fourteen symptomatic and eleven asymptomatic patients deceased, yielding a worrying residual mortality of 24%, significantly higher than previous reports^29,30^, where a 2–5% mortality was recorded. However, in our study we aimed at focusing on the outcomes in the oldest individuals and patients severely disabled living in nursing homes, thus explaining this substantial difference. Noteworthy, after categorizing LUS score by tertiles, patients with the highest LUS score had two and three-times higher mortality compared to those of the lowest tertile, in symptomatic and asymptomatic patients, respectively. This result is not surprising since various studies have reported the post COVID-19 progression to lung fibrosis in older patients^31,32^, and a recent study by Somani et al.^30^, showed that the most common cause for re-admission at hospital after COVID-19 was respiratory distress, typically in comorbid patients.

Asymptomatic patients reported a lower overall mortality, facing most often a reduced interstitial damage detected by LUS score and decreased levels of inflammatory markers compared to symptomatic counterparts. Indeed, the overall lower mortality of asymptomatic patients largely depends on the fact that the majority were in the lowest tertile with half mortality than those with symptoms. On the other hand, mortality risk of asymptomatic patients overcome that of symptomatic in both the second and third LUS score tertiles (35.3% vs 22.2% and 66.7% vs 48.5%, respectively). One of the most interesting results emerging from our study is the demonstration of the complex interaction between frailty and morpho-functional pulmonary impairment in COVID-19 patients, regardless their clinical presentation. Hence, we recorded a higher mortality in pre-frail and frail patients in both symptomatic and symptomatic patients as compared to robust individuals; this result is in line with a recent multi-centre cohort study reporting an increased likelihood of adverse outcomes in patients with CFS score from 4 to 9^33^. Interestingly, we found that a bilateral diffuse interstitial damage may act as a mortality-multiplying risk factor in frail patients, doubling the 3-months mortality moving from the lowest to the higher LUS score tertile.

In conclusion, the current study offers some important insight into the clinical status of asymptomatic COVID-19 older patients, highlighting the prognostic role of LUS evaluation in these individuals, regardless their clinical presentation. Given that frail patients often show non-typical COVID-symptoms and are the most at risk for poor outcomes, the utilization of LUS could guide the clinician in the decision-making process and direct an early-stage COVID-19 therapeutic management. However, the generalization of these results is subject to certain limitations. Firstly, as previously mentioned, due to a small sample size, the findings might not be conclusive; therefore, further prospective analyses in larger cohorts of older patients are needed to confirm these results in asymptomatic patients. Secondly, the study population accounts for two different clinical settings and, some significant differences between the two populations (i.e., age and ADL score) might be biased. Thirdly, in agreement with the current guidelines, we did not performed HRCT to asymptomatic patients; therefore, despite the exclusion of patients with a known history of chronic interstitial disease or pulmonary fibrosis, we cannot exclude an overdiagnosis of COVID-19 pneumonia in asymptomatic subjects, being the B-lines an indirect, non-specific sign of interstitial involvement. Notwithstanding, in the concordance analysis carried out in symptomatic patients, we confirmed a high proportion of agreement between the two techniques.

## Conclusions

The current study targets on the usefulness of a LUS-based follow-up in the frailest older patients with a recent SARS-CoV2 infection regardless symptoms or clinical presentation, and it brings attention on possible drawbacks of the traditional classification of “asymptomatic or pre-symptomatic SARS-CoV2 infection” in the advanced age. Indeed, signs of acute interstitial injury were detected by lung ultrasound in more than two-thirds of older asymptomatic patients. Moreover, mortality rate progressively raised with the increasing degree of interstitial pulmonary involvement, especially in pre-frail and frail patients. These findings raise a warning regarding the actual clinical status of asymptomatic older subjects and suggest bed side ultrasound lung examination as an effective tool to guide clinical workup and patients’ care.

## Supplementary Information

Supplementary Table S1.

Supplementary Figure S1.

Supplementary Figure S2.

## Data Availability

The datasets used and/or analysed during the current study are available from the corresponding author on reasonable request.
