# Supplementary material for: Usefulness of lung ultrasound for selecting asymptomatic older patients with COVID 19 pneumonia
Source: Sci Rep. 2021 Nov 24;11:22892. doi: 10.1038/s41598-021-02275-2 (PMC8613196; doi:10.1038/s41598-021-02275-2)
Supplement: Supplementary file 1 — Supplementary Table S1. [file 41598_2021_2275_MOESM1_ESM.docx]

**Supplemental material legend**

**Supplemental Figure S1a:** High-resolution CT of COVID-19 pneumonia in a 78-years-old symptomatic woman admitted to the geriatric COVID-19 unit, showing bilateral ground glass opacities and posterior pulmonary consolidations.

**Supplemental Figure S1b:** The lung ultrasound examination performed within 12 hours showed an irregular thickened pleural line (white arrow) with discrete and confluent B-lines occupying >50% of the screen (asterisks), consistent with a LUS score 2p for the scanned zone.

**Supplemental Figure S2**: LUS score for monitoring aeration scanning scheme (adapted from Bouhemad *et al.* ^20^)

**Supplemental Table 1: Study population characteristics according to Lung Ultrasound (LUS) Tertiles**

|  | **Whole cohort**  **(N=110)** | **LUS Tertile 1** | **LUS Tertile 2** | **LUS Tertile 3** | **p-value** |
| --- | --- | --- | --- | --- | --- |
| **Age mean, y, (ds)**  Asymptomatic  Symptomatic | 84.2(8.8)  80.8(10.8) | 84.4(9.8)  72.7(18.2) | 84.1(7.9)  82.5(5.6) | 83.7(5.5)  83.0 (7.4) | 0.54  0.15 |
| **Females (%)**  Asymptomatic (%)  Symptomatic (%) | 31/46 (67.4)  24/64 (37.5) | 18 (58.0)  4 (16.7) | 11(35.5)  10(41.7) | 2(6.5)  10(41.7) | 0.80  0.62 |
| **Hs-CRP**  **[mg/dL, mean (ds)]**  Asymptomatic  Symptomatic | 2.7(2.5)  8.9(7.7) | 2.1(2.6)  4.3(6.2) | 3.5(2.3)  8.5(8.0) | 5.3(2.5)  11.1(7.5) | **0.019**  **0.001** |
| **Overall mortality**  **Asymptomatic**   - - Frail (%)   - Pre-Frail (%)   - Robust (%)   **Symptomatic**   - - Frail (%)   - Pre-Frail (%)   - Robust (%) | **13/46 (28.3)**  7/21 (33.3)  3/14 (17.6)  1/8 (17.3)  **23/64 (35.9)**  10/22 (54.5)  3/7 (42.9)  8/35 (34.8) | **4/26 (11.5)**  4/11 (36.3)  0/9 (0)  0/6 (0)  **3/13 (23.1)**  1/4 (25.0)  0 (0)  2/9 (22.2) | **6/17 (35.3)**  4/10 (40.0)  1/5 (20.0)  1/2 (50.0)  **4/18 (22.2)**  2/7(28.6)  0/1(0)  2/10(20.0) | **2/3 (66.7)**  1/1 (100)  1/2 (50.0)  0 (0)  **16/33 (48.5)**  9/11(81.8)  3/6(50.0)  4/16(25.0) | **0.013**  0.057 |

Data are expressed as mean ± standard deviation and number (%) as appropriate. Significant *p* values are marked in bold. HS-CRP: High-sensitivity C-Reactive Protein.
