# Supplementary figures and images for: Usefulness of lung ultrasound for selecting asymptomatic older patients with COVID 19 pneumonia
Source: Sci Rep. 2021 Nov 24;11:22892. doi: 10.1038/s41598-021-02275-2 (PMC8613196; doi:10.1038/s41598-021-02275-2)

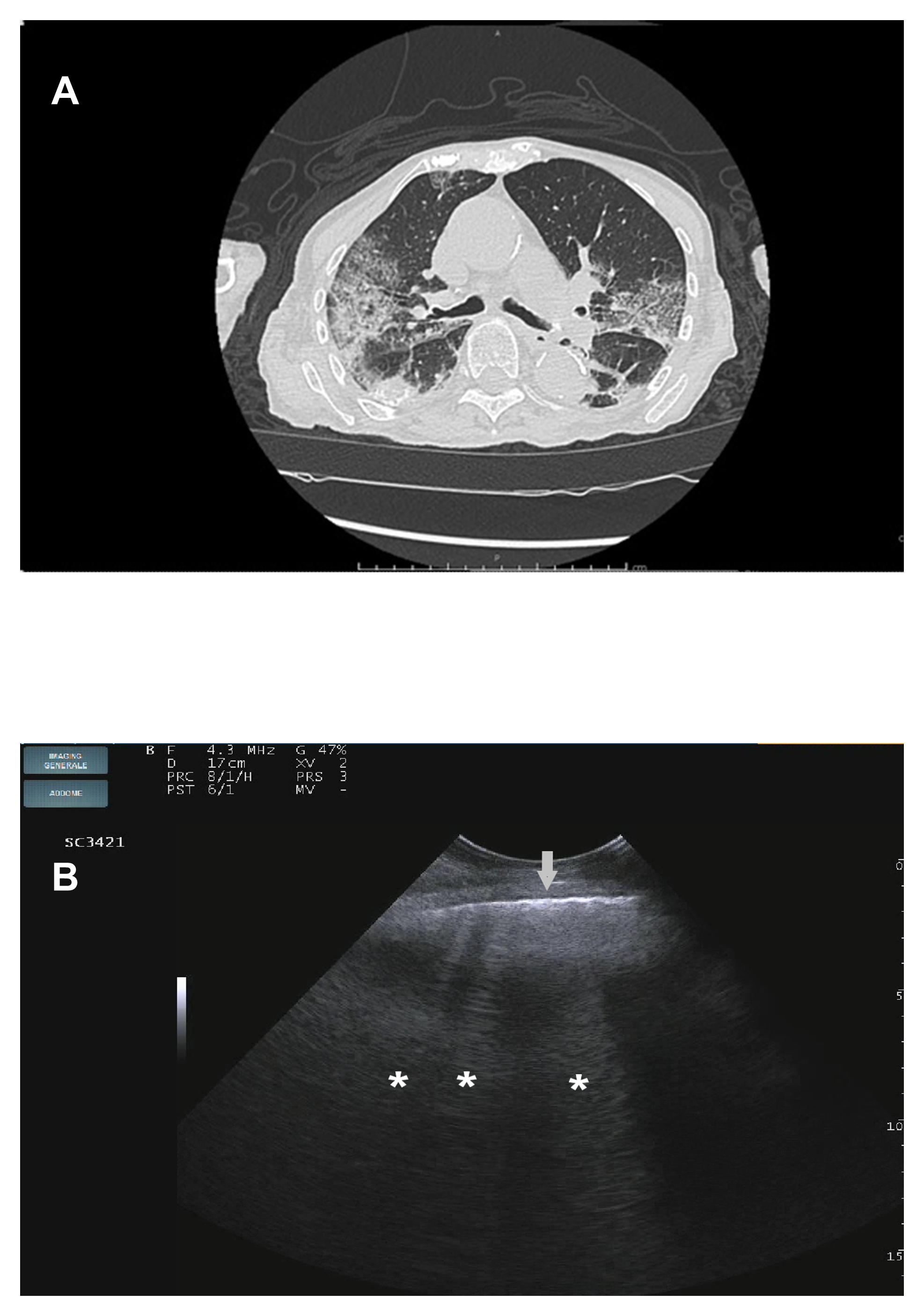

Supplement: Supplementary file 2 — Supplementary Figure S1. [file 41598_2021_2275_MOESM2_ESM.tif]

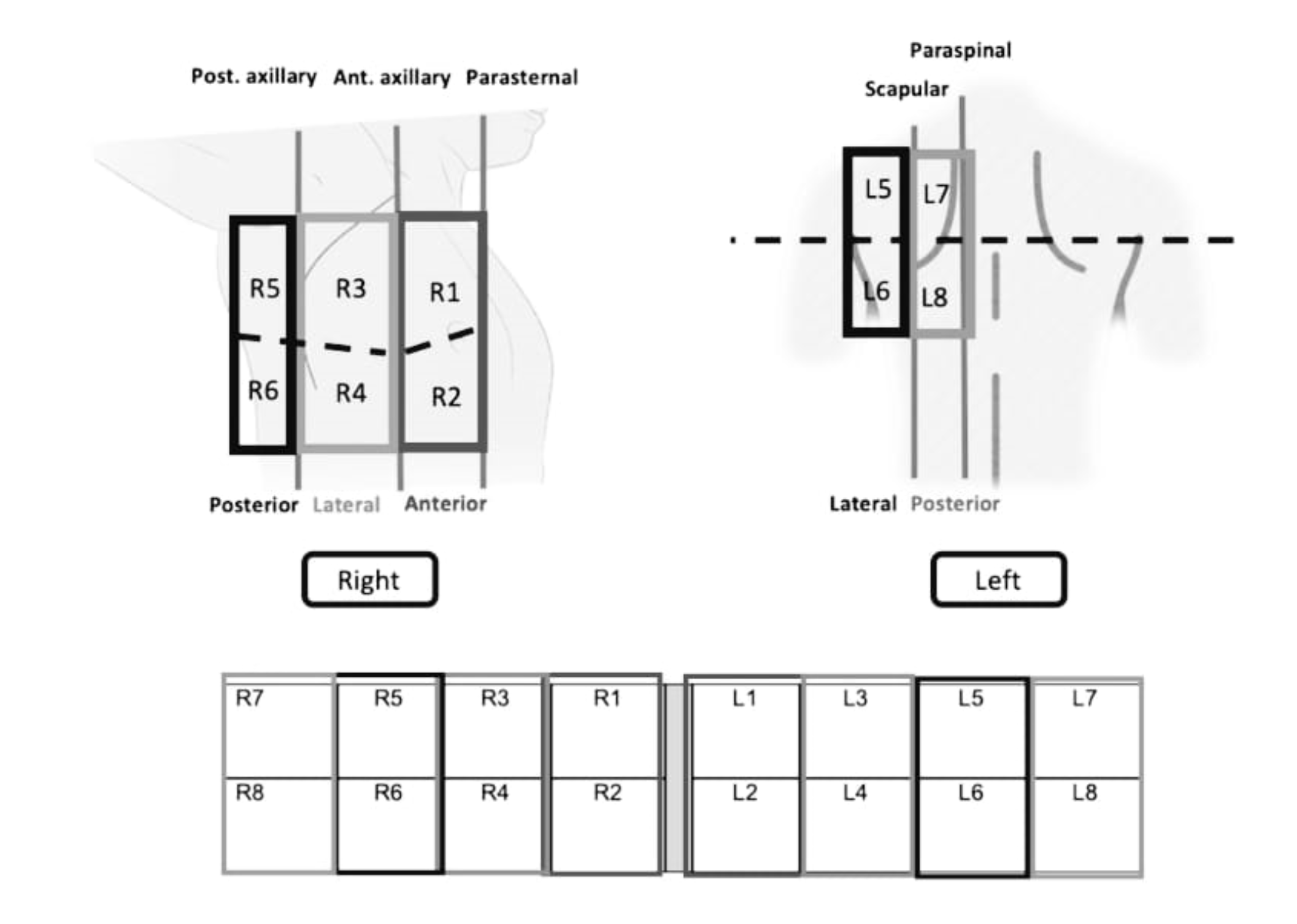

Supplement: Supplementary file 3 — Supplementary Figure S2. [file 41598_2021_2275_MOESM3_ESM.tif]
